# Neonatal infections: Case definition and guidelines for data collection, analysis, and presentation of immunisation safety data^☆^

**DOI:** 10.1016/j.vaccine.2016.03.046

**Published:** 2016-12-01

**Authors:** Stefania Vergnano, Jim Buttery, Ben Cailes, Ravichandran Chandrasekaran, Elena Chiappini, Ebiere Clark, Clare Cutland, Solange Dourado de Andrade, Alejandra Esteves-Jaramillo, Javier Ruiz Guinazu, Chrissie Jones, Beate Kampmann, Jay King, Sonali Kochhar, Noni Macdonald, Alexandra Mangili, Reinaldo de Menezes Martins, César Velasco Muñoz, Michael Padula, Flor M. Muñoz, James Oleske, Melvin Sanicas, Elizabeth Schlaudecker, Hans Spiegel, Maja Subelj, Lakshmi Sukumaran, Beckie N. Tagbo, Karina A. Top, Dat Tran, Paul T. Heath, The Brighton Collaboration Neonatal Infections Working Group

**Affiliations:** a Paediatric Infectious Diseases Research Group, Institute for Infection and Immunity, St George's University of London, UK; b Monash Medical Centre, Clayton, Victoria 3168, Australia; c Madras Medical College, India; d Meyer Children's Hospital, Florence University, Italy; e Enhanced Vigilance Systems, UK; f University of the Witwatersrand, Wits Health Consortium, DST/NRF Respiratory and Meningeal Pathogens Research Unit (RMPRU), South Africa; g Fundação de Medicina Tropical Dr. Heitor Vieira Dourado, Brazil; h Global Pharmacovigilance, Sanofi Pasteur, USA; i GSK Vaccines, Wavre, Belgium; j Imperial College, London, UK; k MRC Unit, The Gambia; l Global Healthcare Consulting, India; m Departments of Paediatrics, Dalhousie University, Halifax, NS, Canada; n Novartis Vaccine/GSK Vaccines, USA; o Bio Manguinhos/Fiocruz, Brazil; p ISGlobal, Barcelona Ctr. Int. Health Res. (CRESIB), Universitad de Barcelona, Spain; q Division of Neonatology, The Children's Hospital of Philadelphia and University of Pennsylvania, USA; r Baylor College of Medicine, Houston, TX, USA; s Department of Pediatrics, Rutgers – New Jersey Medical School, Newark, NJ, USA; t Bill & Melinda Gates Foundation, Seattle, WA, USA; u Division of Infectious Diseases, Global Health Center, Cincinnati Children's Hospital Medical Center, USA; v Henry Jackson Foundation, Bethesda, MD, USA; w National Institute of Public Health (NIJZ), Slovenia; x Centers for Disease Control and Prevention, Division of Pediatric Infectious Diseases – Emory University School of Medicine, USA; y Institute of Child Health, University of Nigeria Teaching Hospital, Nigeria; z Division of Infectious Diseases, Department of Paediatrics, The Hospital for Sick Children, University of Toronto, Canada

**Keywords:** Neonatal infections, Sepsis, Meningitis, Bacteremia, Possible bacterial infection, Pneumonia, Bronchiolitis, Perinatal, Newborn, Adverse event, Immunisation, Guidelines, Case definition

## Abstract

Maternal vaccination is an important area of research and requires appropriate and internationally comparable definitions and safety standards. The GAIA group, part of the Brighton Collaboration was created with the mandate of proposing standardised definitions applicable to maternal vaccine research. This study proposes international definitions for neonatal infections.

The neonatal infections GAIA working group performed a literature review using Medline, EMBASE and the Cochrane collaboration and collected definitions in use in neonatal and public health networks. The common criteria derived from the extensive search formed the basis for a consensus process that resulted in three separate definitions for neonatal blood stream infections (BSI), meningitis and lower respiratory tract infections (LRTI). For each definition three levels of evidence are proposed to ensure the applicability of the definitions to different settings.

Recommendations about data collection, analysis and presentation are presented and harmonized with the Brighton Collaboration and GAIA format and other existing international standards for study reporting.

## 1 Preamble

### 1.1 Need for developing case definitions and guidelines for data collection, analysis, and presentation for neonatal infections as an adverse event following immunisation

Considering the enormous public health benefit that can potentially be derived by vaccinating women in pregnancy to protect their newborns against specific infections, it is now imperative to establish safety and efficacy standards in this area. This includes the need to develop definitions for neonatal infections. Such definitions need to be flexible enough to reflect changes in the pattern of infections that may occur following vaccination and to include infections as possible adverse events [1], [2]. Considering that vaccination may delay the onset of infections from the neonatal period to later in infancy, the definitions also need to be applicable to the young infant.

Providing standardised definitions of neonatal infections is equally relevant for global efforts to address child mortality since the majority of deaths in children less than five years now occur in the neonatal period and neonatal infections are the third most common cause of death in newborns [3]. The majority of deaths occur in low and middle-income countries (LMIC) and therefore standardised definitions for global use must specifically reflect the needs of LMICs. Global deaths from neonatal sepsis and other infections were estimated to be 328,000 and 342,000 in 1990 and 2013, respectively (age-standardised death rates 4.7 and 4.9 per 100,000, respectively) [4]. The other most common types of fatal neonatal infections in 2013 were lower respiratory infections (196,500 deaths), diarrhoeal diseases (44,800), tetanus (26,000), meningitis (20,600), and malaria (16,800) [4].

A variety of definitions for neonatal infections have been proposed and applied in both community and hospital studies (for example from the Young Infant Clinical Study Group (YICSG)) [5], or as part of verbal autopsy studies [6].

In high-income countries, neonatal intensive care has advanced dramatically over the last decades. Neonatal infections cause a significant burden of morbidity and mortality in the extremely preterm population in these settings. As a result, neonatal networks around the world have produced many case definitions for infections, especially focusing on preterm infants. The better known case definitions are from the National Institute of Child Health and Human Development Neonatal Research Network (NICHD) [7], Australian and New Zealand Neonatal Network (ANZNN) (https://npesu.unsw.edu.au/data-collection/australian-new-zealand-neonatal-network-anznn), European Neonatal Network (ENN) [8], the Vermont-Oxford-Network (VON) (https://public.vtoxford.org) and the neonatal infection network (neonIN; www.neonin.org.uk). Some infectious disease networks have focused specifically on healthcare-associated infections, such as neoKISS [9]. With a similar drive to monitoring hospital associated infections, other organisations such as the Centers for Diseases Control (CDC) [10], the European Centre for Disease Control (ECDC) (http://ecdc.europa.eu/en/healthtopics/Healthcare-associated_infections/point-prevalence-survey/Pages/Point-prevalence-survey.aspx) and the European Medicine Agency (EMA) (http://www.ema.europa.eu/docs/en_GB/document_library/Report/2010/12/WC500100199.pdf) have proposed yet more neonatal infection definitions.

In the neonatal period, the immaturity of the immune system, particularly in premature infants, confers distinctive clinical, physical and outcome characteristics to infections compared with other age groups: neonates are more vulnerable to a broad range of pathogens, including those of generally low virulence such as *Listeria*, paraechoviruses or *Candida*. Different pathogens such as bacteria, viruses, fungi or parasites often present in a clinically indistinguishable pattern in neonates, and localised infections may present with systemic signs making the clinical diagnosis difficult and often impossible without imaging confirmation and/or laboratory support. Moreover, a number of non-infectious syndromes, such as respiratory distress syndrome in the premature infant, inborn errors of metabolism and congenital malformations such as serious cardiac anomalies, have initial clinical presentations similar to severe infections [11].

Even when laboratory tests are available, diagnostic tools to guide clinicians are limited. Traditional blood culture methods lack sensitivity, particularly in neonates where only small samples can be obtained. This leads to a high number of negative results, leaving a large percentage of bacterial infections microbiologically unconfirmed [12]. Whilst the diagnosis of some entities such as HIV and CMV has benefited from the use of novel PCR-based molecular diagnostic tools, this has not happened for all neonatal infections. Interpretation of molecular results from non-sterile samples, such as nasopharyngeal aspirates, can be problematic [13].

The lack of a standardised clinical or laboratory diagnosis for neonatal infections explains the heterogeneity in the neonatal infection definitions in current use, particularly for probable bloodstream infections [14].

There is currently no uniformly accepted definition of neonatal infections following immunizations. However, the development of standardised definitions is now essential in order to facilitate comparability of data and outcomes across clinical trials and epidemiological surveillance studies in which women have received vaccines in pregnancy as well as other clinical trials and interventions aimed at reducing neonatal morbidity and mortality.

### 1.2 Methods for the development of the case definition and guidelines for data collection, analysis, and presentation for neonatal infections as an adverse event following immunisation

Following the process described in the overview paper [15], the Brighton Collaboration – GAIA: *Neonatal Infections Working Group* was formed in 2015 and included members with clinical, academic, public health, and vaccine industry background. The composition of the working and reference group as well as results of the web-based survey completed by the reference group with subsequent discussions in the Working Group can be viewed at: http://www.brightoncollaboration.org/internet/en/index/working_groups.html.

To guide the decision-making for the case definition and guidelines, a literature search was performed using Medline, Embase and the Cochrane Libraries, the search terms are available in Appendix 1.

The search resulted in the identification of 4422 references. Only references with full abstracts (in English language) were included. All abstracts were screened for possible reports of neonatal infections. 1205 articles with potentially relevant material were reviewed in more detail. This review resulted in a detailed summary of 432 articles, including information on the diagnostic criteria or case definitions used. Case reports, editorials and letters were excluded. Where relevant a description of the vaccine used, the time interval since immunisation, and any other symptoms were extracted. Multiple key references were hand searched and definitions from existing neonatal networks, infection surveillance networks and websites of public health organisations such as the Centers for Disease Control (CDC), the European Centre for Disease Control (ECDC) and the European Medicine Agency (EMA) were also searched for neonatal and perinatal infection definitions.

Across the different manuscripts selected, a large number of definitions were found with a variable number and type of clinical, laboratory and microbiological criteria. The quality of the manuscripts was heterogeneous but this review did not grade the evidence as it was not considered to be relevant for the task of extracting the definitions used.

The definitions from the manuscripts were extracted and entered into spreadsheets listing clinical, laboratory and radiological criteria by 14 members of the group independently and then reviewed for consistency by the coordinator (SV). The data were separated according to the syndrome described: sepsis, meningitis and respiratory tract infections and congenital infections. Percentages of the clinical and laboratory indicators were calculated. The syndromes were not separated according to single pathogens or class of pathogens.

The data extracted from the published literature were collected recognizing the limitation that each study reported different data and definitions for the clinical or laboratory signs and these were not always specified nor clearly described. The studies from neonatal units in high-income countries were reporting both clinical and laboratory confirmed infections while community studies from middle- or low-income countries used mostly clinical definitions. This heterogeneity made data extraction a somewhat subjective exercise. Proposed definitions for specific congenital infections were also discussed, but were eventually excluded from this guideline and recommended for consideration as a specific group of definitions for a future Brighton collaboration Working Group.

The results of this work were presented to the Working Group together with the standard definitions currently in use from the aforementioned networks and the group discussed the definitions in a series of teleconferences until consensus was obtained.

### 1.3 Rationale for selected decisions about the case definition of neonatal infections as an adverse event following immunisation

For the purpose of this guideline the term “infection” includes neonatal bacteraemia and sepsis (of early or late onset), meningitis, pneumonia and other respiratory infections such as bronchiolitis, caused by bacteria, parasites, viruses or fungi. Localised eye and ear infections were excluded from these guidelines as were encephalitis, urinary tract infections and intestinal infections. The term “neonatal” includes infants from birth (day 0) up to and including 28 postnatal days.

The term “neonatal infection” was chosen to include different infection syndromes during the neonatal period (proven blood stream infections, probable blood stream infections, meningitis and respiratory tract infections).

Ultimately, the group reached agreement on 3 separate definitions for neonatal infections, each with 3 or more diagnostic levels. It is important to emphasise that within the definition context, however, the diagnostic levels must not be misunderstood as reflecting different grades of clinical severity. They instead reflect diagnostic certainty. The case definition has been formulated such that the Level One definition is highly specific for the condition. As maximum specificity normally implies a loss of sensitivity, one or two additional diagnostic levels have been included in the definition, offering a stepwise increase of sensitivity from level one to level three, while retaining an acceptable level of specificity at all levels. In this way it is hoped that all possible cases of neonatal infections can be captured, regardless of the setting or population in which they are being assessed. This is of particular relevance in LMICs where the resources available to assess events, e.g. laboratory facilities, may be more limited.

#### 1.3.1 Rationale for individual criteria or decisions made related to the case definition

##### 1.3.1.1 Neonatal invasive blood stream infections

The GAIA neonatal infections Working Group included in level 1 the microbiological confirmation of infection as this is the recognised diagnostic gold standard. It was decided to use the term “validated” method of identification because it was recognised that this is a rapidly changing field, especially with regard to molecular tests. It is hoped that this will allow the definition to be as inclusive as possible as these methods continue to advance.

The group opted to include a list of organisms commonly considered non-pathogenic (often called “skin commensals”), but still capable of causing opportunistic infections in certain situations, for example, in the presence of central lines, as well as a list of recognised pathogens in order to reduce uncertainty and differences in reporting.

The number of clinical criteria was chosen by reviewing the available definitions in the literature and by consensus. It was decided to include a level 3 definition based solely on clinical signs and taken from a systematic review of studies that reported clinical signs predictive of severe illnesses or mortality in young infants aged 0–59 days, endorsed by the World Health Organization (WHO) [16]. The limited set of clinical signs for which extensive evidence supporting their value exists was reported to have high sensitivity and reasonable specificity. This ensures that the case definition has relevance in all populations and settings.

With regard to the criterion of abnormal white cell count (WCC), it is recognised that ethnic variations exist, for example many African Americans have a WCC that is persistently below the normal range for people of European descent, a condition called “benign ethnic neutropenia” [17]. This should be considered when evaluating a case.

##### 1.3.1.2 Neonatal meningitis

As above, the GAIA neonatal infections Working Group included in level 1 the microbiological confirmation of infection as this is the recognised diagnostic gold standard.

In recognition that delays in undertaking a lumbar puncture may mean that antibiotics have already been given before CSF is obtained, which may make microbiological confirmation less likely, the group included a definition based on the presence of CSF pleocytosis. CSF pleocytosis was defined as ≥20 cells/mm^3^ for ≤28 day-olds and ≥10 cells/mm^3^ for 29–89 day-olds based on data from large studies [18], [19] with no adjustment made for traumatic taps [20].

##### 1.3.1.3 Respiratory tract infections (RTI)

The GAIA neonatal infections Working Group provided a single definition for RTI which aimed to include bacterial, fungal and viral pathogens to allow ease of use. The different pathophysiology of viral and bacterial or fungal infections is reflected in the use of diagnostic imaging. Radiographic features (e.g. lobar infiltrate) were accepted without microbiological confirmation for bacterial and fungal infections, but viral low respiratory tract infections required laboratory confirmation, even in the presence of X-ray findings consistent with a viral diagnosis.

The number of clinical criteria chosen arose from the consensus of the group after careful review of available evidence and current definitions in use.

The Working Group were aware of the proposed WHO candidate case definitions for RSV vaccine efficacy trials and believe that both sets of guidelines are consistent.

#### 1.3.2 Influence of treatment on fulfilment of case definitions [21]

In the context of infection a response to antimicrobial treatment might be considered towards fulfilment of the neonatal infections case definition. However, the Working Group decided against this. A treatment response or its failure is not in itself diagnostic and may depend on variables such as clinical status, time to initiation of treatment, other clinical parameters and for many infections, particularly viral, no treatment is currently available.

Inflammatory markers were included although it was recognised that viral infections often are not accompanied by an inflammatory response and newborns often do not present a strong inflammatory response, particularly extremely preterm infants.

#### 1.3.3 Timing post immunisation

Specific time frames for onset of symptoms following immunisation are not included because there are many factors that may influence the impact of vaccination in pregnancy on events in the newborn period. Such factors include the vaccine given, the length of gestation at vaccination of the mother and at birth, the presence of pre-existing immunity and concomitant illnesses in the newborn.

We postulate that a definition designed to be a suitable tool for testing causal relationships requires ascertainment of the outcome independent from the exposure (e.g. immunisations). Therefore, to avoid selection bias, a restrictive time interval from immunisation to the onset of neonatal infections should not be an integral part of such a definition. Instead, where feasible, details of this interval should be assessed and reported as described in the data collection guidelines.

Further, events often occur outside the controlled setting of a clinical trial or hospital. In some settings it may be impossible to obtain a clear timeline of the event, particularly in less developed or rural settings. In order to avoid selecting against such cases, the case definition avoids setting arbitrary time frames.

#### 1.3.4 Differentiation from other (similar/associated) disorders

Using the level 2 or 3 of evidence there is risk that the above definitions will include other neonatal pathologies such as congenital heart diseases or inborn errors of metabolism within the blood stream infections (BSI) and meningitis definitions or even respiratory distress syndrome and transient tachypnea of the newborn in the most premature neonates within the RTI definition. Congenital malformations and inborn error of metabolism are relatively rare events however, and distinction based on clinical response to treatment, laboratory investigations and imaging may be possible in most settings.

### 1.4 Guidelines for data collection, analysis and presentation

The case definition is accompanied by guidelines, which are structured according to the steps of conducting a clinical trial, i.e. data collection, analysis and presentation. Neither case definition nor guidelines are intended to guide or establish criteria for management of ill infants, children, or adults. Both were developed to improve data comparability.

### 1.5 Periodic review

Similar to all Brighton Collaboration case definitions and guidelines, review of the definition with its guidelines is planned on a regular basis (i.e. every three to five years) or more often if needed.

## 2 Case definitions of neonatal infections

### 2.1 Neonatal invasive blood stream infections: bacterial/fungal/viral

Table 1 presents the case definition for neonatal invasive blood Stream Infections: bacterial/fungal/viral.

### 2.2 Bacterial/fungal/viral meningitis

Table 2 presents the case definition for bacterial/fungal/viral meningitis.

### 2.3 Respiratory bacterial/fungal/viral infection

Table 3 presents the case definition for respiratory bacterial/fungal/viral infection.

## 3 Guidelines for data collection, analysis and presentation of neonatal infections

It was the consensus of the Brighton Collaboration GAIA *Neonatal Infections Working Group* to recommend the following guidelines to enable standardised data collection, analysis, and presentation of information regarding neonatal infections in the context of pregnancy vaccination. The availability of information may vary depending upon resources, geographical region, and whether the source of information is a prospective clinical trial, a post-marketing surveillance or epidemiological study, or an individual report of a neonatal infection.

Guidelines for the collection, analysis and presentation of safety data in clinical trials of vaccines in pregnant women are also available and should be referred to for more generic guidance.

### 3.1 Data collection

#### 3.1.1 Source of information/reporter

For all cases and/or all study participants, as appropriate, the following information should be recorded:

(1) Date of report.
(2) Name and contact information of person reporting^2^ and/or diagnosing the event as specified by country-specific data protection law.
(3) Name and contact information of the investigator responsible for the subject, as applicable.
(4) Relation to the patient (e.g. immuniser [clinician, nurse], family member [indicate relationship], other).

#### 3.1.2 Vaccinee/control

For all cases and/or all study participants, as appropriate, the following information should be recorded:

##### 3.1.2.1 Demographics

(5) Case/study participant identifiers (e.g. first name initial followed by last name initial) or code (or in accordance with country-specific data protection laws).
(6) Date of birth, age, and sex. With neonatal data disaggregated from older infants.
(7) Gestational age, birth weight and methods used for their assessment.

##### 3.1.2.2 Clinical and immunisation history

For all cases and/or all study participants, as appropriate, the following information should be recorded:

(8) Mother: Maternal history of infections or risk factors for infections (e.g. GBS colonisation, peripartum fever), indication whether any antimicrobials were used in pregnancy or in labour, type and route of administration; underlying diseases/disorders, type of delivery and indicate whether the delivery occurred in a facility or at home, describe obstetric care available in terms of basic or comprehensive; immunisation received in pregnancy with dates, type, batch and reaction for all infections, available serology as applicable, any other medications use during pregnancy including non prescription medications.
(9) Newborn: report whether the newborn was admitted to hospital and the type of facility (e.g. emergency department, ward, neonatal unit) or was in the community. Indicate the level of neonatal care available (e.g. ventilator support) and give the type of neonatal care staff available and their level of training, Indicate the presence of central lines, whether the newborn received surgical interventions and their type.
(10) Newborn: Report the medication history (other than treatment for the event described) including prescription and non-prescription medication as well as medication, topical treatments, parenteral nutrition or treatment with long half-life or long-term effect (e.g. immunoglobulins, blood transfusion and immunosuppressants).
(11) Facility: indicate whether microbiology laboratory investigations are available and describe the methods used for bacterial identification or the molecular techniques used to identify organisms viral, fungal, parasitic or bacterial. Give an indication of the quality control in place. Indicate whether biochemistry, haematology and radiology facilities are available.
(12) Immunisation history (i.e. previous immunizations and any adverse event following immunisation (AEFI)), in particular occurrence of neonatal infection after a previous immunisation.

#### 3.1.3 Details of the immunisation

For all study participants, as appropriate, the following information about pregnancy vaccination should be recorded:

(13) Date and time of immunisation(s), gestational age at the time of immunisation. Context of immunisation (routine clinic, outbreak situation, clinical trial, etc.)
(14) Description of vaccine(s) (name of vaccine, manufacturer, lot number, dose (e.g. 0.25 mL, 0.5 mL, etc.) and number of dose if part of a series of immunizations against the same disease).
(15) The anatomical sites (including left or right side) of all immunizations (e.g. vaccine A in proximal left lateral thigh, vaccine B in left deltoid).
(16) Route and method of administration (e.g. intramuscular, intradermal, subcutaneous, and needle-free (including type and size), oral, intranasal, other injection devices).
(17) Needle length and gauge.

#### 3.1.4 The adverse event

(18) For all cases at any level of diagnostic certainty and for reported events with insufficient evidence, the criteria fulfilled to meet the case definition should be recorded.
  Specifically document:
(19) Clinical description of signs of neonatal infection and if there was confirmation of the infection (i.e. positive identification using validated method).
(20) Date/time of onset,^3^ first observation^4^ and diagnosis,^5^ end of episode^6^ and final outcome.^7^
(21) Concurrent signs and diseases.
(22) Measurement/testing
  • Values and units of routinely measured parameters (e.g. temperature, blood pressure) – in particular those indicating the severity of the event;
  • Method of measurement (e.g. type of thermometer, oral or other route, duration of measurement, etc.);
  • Results of laboratory examinations, surgical and/or pathological findings and diagnoses if present.
(23) Treatment given for neonatal infection, especially antimicrobials, including which antimicrobials (e.g. antibiotics, antivirals, immunoglobulins), dosing and duration of treatment.
(24) Outcome^6^ at last observation.
(25) Objective clinical evidence supporting classification of the event as “serious”^8^
(26) Exposures from 24 h before and after immunisation (e.g. food, environmental) considered potentially relevant to the reported event.

#### 3.1.5 Miscellaneous/general

(27) The duration of surveillance for neonatal infection should be predefined based on
  • Biologic characteristics of the vaccine e.g. live attenuated versus inactivated component vaccines;
  • Biologic characteristics of the vaccine-targeted disease;
  • Biologic characteristics of neonatal infection including patterns identified in previous trials (e.g. early-phase trials); and
  • Biologic characteristics of the vaccinee (e.g. nutritional status, underlying disease like immunosuppressing illness).
(28) The duration of follow-up reported during the surveillance period should be predefined likewise. It should aim to continue to resolution of the event.
(29) Methods of data collection should be consistent within and between study groups, if applicable.
(30) Follow-up of cases should attempt to verify and complete the information collected as outlined in data collection guidelines.
(31) Investigators of patients with neonatal infection should provide guidance to reporters to optimise the quality and completeness of information provided.
(32) Reports of neonatal infection should be collected throughout the study period regardless of the time elapsed between immunisation and the adverse event. If this is not feasible due to the study design, the study periods during which safety data are being collected should be clearly defined.

### 3.2 Data analysis

The following guidelines represent a desirable standard for analysis of data on neonatal infections to allow for comparability of data, and are recommended in addition to the data analysed for the specific study question and setting.

Reported events should be classified in one of the following five categories including the three levels of diagnostic certainty. Events that meet the case definition should be classified according to the levels of diagnostic certainty as specified in the case definition. Events that do not meet the case definition should be classified in the additional categories for analysis.

**Event classification in 5 categories**^9^

• Event meets case definition
  (1) Level 1: Criteria as specified in the neonatal infections case definition (separately for BSI, meningitis and RTI)
  (2) Level 2: Criteria as specified in the neonatal infection case definition (separately for BSI, meningitis and RTI)
  (3) Level 3: Criteria as specified in the neonatal infections case definition (separately for BSI, meningitis and RTI)
• Event does not meet case definition
  *Additional categories for analysis*
  (4) Reported neonatal infection (separately for BSI, meningitis and RTI) with insufficient evidence to meet the case definition^10^
  (5) Not a case of neonatal infection^11^ neither BSI, meningitis or RTI

The interval between maternal immunisation and reported neonatal infection could be defined as the interval from the date/time of immunisation to the date/time of onset^2^ of the first signs consistent with the definition. The timing of onset of a neonatal infection may be defined by the age of the infant at the time of onset using specific periods of infancy as follows:

Periods of infancy for age of clinical recognition of a neonatal infection

**Table:** 

Time period	Days
Prenatal	<Day 1 of life
Neonatal^a^	1–27^b^
Early neonatal^a^	1–6^b^
Late neonatal^a^	7–27
Post neonatal	28–364

a Use either Neonatal or divide into early neonatal and late neonatal.

b Day 1 = first 24 h of life.

The duration of a possible neonatal infection could be analysed as the interval between the date/time of onset^2^ of the first signs consistent with the definition and the end of episode^5^ and/or final outcome^6^. Whatever start and ending are used, they should be used consistently within and across study groups.

If more than one measurement of a particular criterion is taken and recorded, the value corresponding to the greatest magnitude of the adverse experience could be used as the basis for analysis. Analysis may also include other characteristics like qualitative patterns of criteria defining the event.

The distribution of data (as numerator and denominator data) could be analysed in predefined increments (e.g. measured values, times), where applicable. Increments specified above should be used. When only a small number of cases is presented, the respective values or time course can be presented individually.

Data on neonatal infections obtained from neonates born to women vaccinated during pregnancy should be compared with those obtained from an appropriately selected and documented control group(s) or known background rates of neonatal infections in comparable populations, and should be analysed by study arm and dose where possible, e.g. in prospective clinical trials.

### 3.3 Data presentation

These guidelines represent a desirable standard for the presentation and publication of data on neonatal infections following maternal immunisation to allow for comparability of data, and are recommended as an addition to data presented for the specific study question and setting. Additionally, it is recommended to refer to existing general guidelines for the presentation and publication of randomised controlled trials, systematic reviews, and meta-analyses of observational studies in epidemiology (e.g. statements of Consolidated Standards of Reporting Trials (CONSORT), of Improving the quality of reports of meta-analyses of randomised controlled trials (QUORUM), and of Meta-analysis Of Observational Studies in Epidemiology (MOOSE), respectively) and the Strengthening the Reporting of Observational Studies in Epidemiology (STROBE) and Strengthening the Reporting of Observational Studies in Epidemiology for Newborn Infection (STROBE-NI) guidelines (Fitchett, in press) (http://www.equator-network.org).

All reported events of neonatal infections should be presented according to the categories listed above.

Data on possible neonatal infections events should be presented in accordance with data collection guidelines and data analysis guidelines.

Terms to describe neonatal infection such as “low-grade”, “mild”, “moderate”, “severe” or “significant” are highly subjective, prone to wide interpretation, and should be avoided, unless clearly defined.

Data should be presented with numerator and denominator (n/N) (and not only in percentages), if available. It should be clear if the denominator represents a population denominator (live births) or neonates admitted to a facility. The source of the denominator data should be reported and calculations of estimate described (e.g. manufacturer data like total doses distributed, reporting through Ministry of Health, coverage/population based data, etc.).

The incidence of cases in the study population should be presented and clearly identified as such in the text.

If the distribution of data is skewed, median and interquartile range are usually the more appropriate statistical descriptors than the mean. However, the mean and standard deviation should also be provided.

Any publication of data on neonatal infection should include a detailed description of the methods used for data collection and analysis as possible. It is essential to specify:

• The study design;
• The method, frequency and duration of monitoring for neonatal infection;
• The trial profile, indicating participant flow during a study including drop-outs and withdrawals to indicate the size and nature of the respective groups under investigation;
• The type of surveillance (e.g. passive or active surveillance);
• The characteristics of the surveillance system (e.g. population served, mode of report solicitation);
• The search strategy in surveillance databases;
• Comparison group(s), if used for analysis;
• The instrument of data collection (e.g. standardised questionnaire, diary card, report form);
• Whether the day of immunisation was considered “day one” or “day zero” in the analysis;
• Whether the date of onset^2^ and/or the date of first observation^3^ and/or the date of diagnosis^4^ was used for analysis; and
• Use of this case definition, in the abstract or methods section of a publication.^12^

## Figures and Tables

**Table 1:** Neonatal invasive blood stream infections: bacterial/fungal/viral.

LEVEL 1	LEVEL 2	LEVEL 3 [22]
Recognised pathogen^a^ identified using a validated method and from a normally sterile site^b^ If an organism normally considered non-pathogenic is isolated from blood cultures^a^: Level 1 requires its identification from at least 2 blood cultures taken from two different sites, or at 2 different times, PLUS 1 of the criteria as per level 2 of evidence	Not meeting Level 1 of evidence **AND** 3 or more criteria: • Temperature ≥37.5 °C or <35.5 °C^f^ • Tachycardia^d^ or new or more frequent episodes of bradycardia^d^ • New or more frequent episodes of apnea^d^ or increased oxygen requirement or increased requirement for ventilatory support • Lethargy or moving only when stimulated or hypotonia or irritability • Difficulty in feeding or abdominal distention • Pallor or poor perfusion^d^or hypotension^d^ • Abnormal White Cell Count^d^ or I/T ratio >0.2 • Abnormal platelet count d • Increased^e^ inflammatory markers (CRP, procalcitonin) • Metabolic acidosis as defined by a base excess (BE)^d^	Not meeting Level 1 or 2 of evidence **AND** 2 or more of the following criteria: • Temperature ≥37.5 °C or <35.5 °C^f^ • Tachypnea^d^ or severe chest indrawing or grunting or cyanosis • Change in level of activity • History of feeding difficulty • History of convulsions

a See list of pathogens and non-pathogens in Appendix 1.

b Sterile site: blood, sterile urine (catheter urine or supra-pubic aspirate), pleural fluid, ascitic fluid, broncho-alveolar lavage, bone biopsy, synovial fluid.

d *Definitions*: Apnea: pause in breathing >20 s; CRP or calcitonin levels above the local normal standards; Tachypnea/fast breathing: respiratory rate >60 breaths per minute; Tachycardia: heart rate >180 beats per minute; Bradycardia: heart rate <100 beats per minute; c Poor perfusion: CRT >2. d 4000 or >20,000 × 10^9^ cells/L; Low Platelets/Thrombocytopenia: <100,000 × 10^9^/L; Metabolic acidosis: <−10 mmol/L (−10 mEq/L)

e Increased according to locally defined and validated reference ranges.

f Also refer to Brighton collaboration case definition for fever [23].

**Table 2:** Bacterial/fungal/viral meningitis.

LEVEL 1	LEVEL 2	LEVEL 3a	LEVEL 3b
Recognised pathogen^a^ identified using a validated method from cerebrospinal fluid (CSF) If an organism normally considered non-pathogenic is identified from the CSF, LEVEL 1 of evidence additionally requires all LEVEL 2 criteria: i.e. CSF pleocytosis AND temperature criteria AND 1 or more clinical criteria	CSF pleocytosis^d^ OR positive IgM antibodies to a specific pathogen in the CSF **AND** Recognised pathogen^a^ identified using a validated method from a normally sterile site^b^ (other than CSF) **AND** Temperature ≥37.5 °C or <35.5 °C^c^ **AND** 1 or more criteria: • History of convulsions • Lethargy or irritability • Coma • Apnea^d^ • Bulging fontanel • Neck stiffness	CSF pleocytosis^d^ **AND** NO d pathogen^a^ identified using a validated method from a normally sterile site^b^ **AND** Temperature ≥37.5 °C or <35.5 °C^c^ **AND** 3 or more criteria: • History of convulsions • Lethargy or irritability • Coma • Apnea^d^ • Bulging fontanel • Neck stiffness	No lumbar puncture done or no sample available **AND** Temperature ≥37.5 °C or <35.5 °C^c^ **AND** 4 or more criteria: • History of convulsions • Lethargy or irritability • Coma • Apnea^d^ • Bulging fontanel • Neck stiffness

a See list of pathogens and non-pathogens in Appendix 1.

b Sterile site: blood, sterile urine (catheter urine or supra-pubic aspirate), pleural fluid, ascitic fluid, broncho-alveolar lavage, bone biopsy, synovial fluid.

c Also refer to Brighton collaboration case definition for fever [23].

d CSF pleocytosis: ≥20 cells/mm^3^ for <28 day-olds and ≥10 cells/mm^3^ for 29–89 day-olds. # i–89 day-olds.

**Table 3:** Respiratory bacterial/fungal/viral infection.

LEVEL 1	LEVEL 2	LEVEL 3 [24], [25]
New or progressive or persistent infiltrate or shadowing or fluid in the intrapleural cavity or interlobar fissure on chest X-ray AND Recognised virus^c^ identified using a validated assay from an upper respiratory sample OR Recognised pathogen^a^ identified using a validated method and from a normally sterile site^b^ **AND 3 or more criteria:** • Temperature ≥37.5 °C or <35.5 °C^e^ • Tachypnea^c^ or Nasal flaring or Chest indrawing or Grunting • Desaturations or increased oxygen requirements or increased ventilator requirements or oxygen saturation <95% • Apneas^c^ • Increased respiratory secretions or Increased suctioning requirements • Cough or wheeze or crepitations • Increased CRP or procalcitonin^d^	New or progressive or persistent infiltrate or shadowing or fluid in the intrapleural cavity or interlobar fissure on chest X-ray **AND 4 or more criteria:** • Temperature ≥37.5 °C or <35.5 °C^e^ • Tachypnea^c^ or Nasal flaring or Chest indrawing or Grunting • Desaturations or increased oxygen requirements or increased ventilator requirements or oxygen saturation <95% • Apneas^c^ • Increased respiratory secretions or Increased suctioning requirements • Cough or wheeze or crepitations • Increased CRP or procalcitonin^d^	**2 or more criteria:** Difficulty in breathing/Tachypnea^c^ • Severe chest indrawing • Nasal flaring • Grunting • Wheezing • Stridor • Fever

a See list of pathogens and non-pathogens in Appendix 1.

b Sterile site: blood, sterile urine (catheter urine or supra-pubic aspirate), pleural fluid, ascitic fluid, broncho-alveolar lavage, bone biopsy, synovial fluid.

c See list of definitions in Table 1.

d Increased according to locally defined and validated reference ranges.

e Also refer to Brighton collaboration case definition for fever [23].

## Footnotes

2 If the reporting centre is different from the vaccinating centre, appropriate and timely communication of the adverse event should occur.

3 The date and/or time of onset is defined as the time post immunisation, when the first sign or symptom indicative for neonatal infection occurred. This may only be possible to determine in retrospect.

4 The date and/or time of first observation of the first sign or symptom indicative for neonatal infection can be used if date/time of onset is not known.

5 The date of diagnosis of an episode is the day post immunisation when the event met the case definition at any level.

6 The end of an episode is defined as the time the event no longer meets the case definition at the lowest level of the definition.

7 E.g. recovery to pre-immunisation health status, spontaneous resolution, therapeutic intervention, persistence of the event, sequelae, death.

8 An AEFI is defined as serious by international standards if it meets one or more of the following criteria: (1) it results in death, (2) is life-threatening, (3) it requires inpatient hospitalisation or results in prolongation of existing hospitalisation, (4) results in persistent or significant disability/incapacity, (5) is a congenital anomaly/birth defect, (6) is a medically important event or reaction.

9 To determine the appropriate category, the user should first establish, whether a reported event meets the criteria for the lowest applicable level of diagnostic certainty, e.g. Level three. If the lowest applicable level of diagnostic certainty of the definition is met, and there is evidence that the criteria of the next higher level of diagnostic certainty are met, the event should be classified in the next category. This approach should be continued until the highest level of diagnostic certainty for a given event could be determined. Major criteria can be used to satisfy the requirement of minor criteria. If the lowest level of the case definition is not met, it should be ruled out that any of the higher levels of diagnostic certainty are met and the event should be classified in additional categories four or five.

10 If the evidence available for an event is insufficient because information is missing, such an event should be categorised as “Reported neonatal infection with insufficient evidence to meet the case definition”.

11 An event does not meet the case definition if investigation reveals a negative finding of a necessary criterion (necessary condition) for diagnosis. Such an event should be rejected and classified as “Not a case of neonatal infection”.

12 Use of this document should preferably be referenced by referring to the respective link on the Brighton Collaboration website (http://www.brightoncollaboration.org).
