# Supplementary material for: Neonatal infections: Case definition and guidelines for data collection, analysis, and presentation of immunisation safety data
Source: Vaccine. 2016 Dec 1;34(49):6038–46. doi: 10.1016/j.vaccine.2016.03.046 (PMC5139809; doi:10.1016/j.vaccine.2016.03.046)
Supplement: Supplementary file 1 [file mmc1.docx]

GAIA Neonatal Infections EMBASE; Run 9.7.2015

1. *infection/ or exp hospital infection/ or exp bloodstream infection/ or exp bacteremia/ or exp catheter infection/

2. exp virus infection/ or *infection/

3. exp septic shock/

4. exp staphylococcal bacteremia/ or exp bacteremia/

5. exp septicemia/ or exp hemorrhagic septicemia/ or exp Viral hemorrhagic septicemia virus/ or exp viral hemorrhagic septicemia/

6. exp bacterial infection/

7. exp central nervous system infection/ or exp bacterial meningitis/

8. *infection/ or exp meningoencephalitis/ or expmeningococcosis/ or exp meningitis/

9. exp meningoencephalitis/

10. exp fungal meningitis/ or exp pneumococcal meningitis/ or exp group B streptococcal meningitis/ or meningitis/ or exp Candida meningitis/ or expHaemophilus meningitis/ or exp bacterial meningitis/ or exp aseptic meningitis/ or exp meningitis vaccine/ or exp virus meningitis/ or exp epidemic meningitis/

11. exp omphalitis/

12. exp brain ventriculitis/

13. exp group B streptococcal pneumonia/ or exp ventilator associated pneumonia/ or exp lobar pneumonia/ or exp Pseudomonas pneumonia/ or exp chlamydial pneumonia/ or exp health care associated pneumonia/ or exp infectious pneumonia/ or exp virus pneumonia/ or exp hospital acquired pneumonia/ or exp bacterial pneumonia/ or exp Streptococcus pneumonia/ or exp pneumonia/ or exp staphylococcal pneumonia/ or exp Escherichia coli pneumonia/

14. exp chest infection/

15. exp bronchiolitis/ or exp viral bronchiolitis/

16. exp osteomyelitis/

17. exp osteomyelitis/ or exp infectious arthritis/ or exp bone infection/ or exp soft tissue infection/

18. exp bacterial arthritis/

19. exp hospital infection/

20. exp urinary tract infection/ or exp Staphylococcus infection/

21. exp vertical transmission/ or exp newborn infection/ or exp Streptococcus infection/ or exp Streptococcus agalactiae/

22. exp Escherichia coli/

23. exp methicillin resistant Staphylococcus aureus/

24. exp Staphylococcus aureus/

25. exp Enterobacter aerogenes/ or exp Enterobacter/ or exp "Enterobacter cloacae subsp. dissolvens"/ or exp Enterobacter cloacae/

26. exp Acinetobacter junii/ or exp Acinetobacter baumannii/ or exp Acinetobacter infection/ or exp Acinetobacter/ or exp Acinetobacter calcoaceticus/ or exp Acinetobacter lwoffii/ or exp Acinetobacter baylyi/ or exp Acinetobacter johnsonii/

27. expKlebsiella pneumoniae infection/ or expKlebsiella vaccine/ or expKlebsiella/ or expKlebsiellaoxytoca/ or expKlebsiella infection/ or expKlebsiella pneumoniae/

28. exp Pseudomonas infection/ or exp Pseudomonas aeruginosa/ or exp Pseudomonas vaccine/ or exp Pseudomonas/ or exp Pseudomonas stutzeri/

29. expSerratia infection/ or expSerratia/ or expSerratiamarcescens/ or expSerratialiquefaciens/

30. exp Listeria monocytogenes/ or exp Listeria/

31. exp Staphylococcus aureus/ or exp bacteremia/ or exp bacterial infection/ or exp coagulase negative Staphylococcus/ or exp hospital infection/ or exp surgical infection/ or exp catheter infection/ or exp Staphylococcus infection/

32. exp bronchiolitis/ or exp respiratory syncytial virus infection/ or exp Respiratory syncytial pneumovirus/ or *infection/

33. exp Human cytomegalovirus/ or exp Cytomegalovirus/ or exp cytomegalovirus infection/ or exp Cytomegalovirus vaccine/

34. exp diphtheria pertussis tetanus Haemophilusinfluenzae type b vaccine/ or exp pertussis vaccine/ or exp diphtheria pertussis poliomyelitis tetanus hepatitis B vaccine/ or exp diphtheria pertussis tetanus hepatitis B vaccine/ or exp pertussis/ or exp diphtheria pertussis poliomyelitis tetanus Haemophilusinfluenzae type b vaccine/ or exp diphtheria pertussis tetanus vaccine/ or exp diphtheria pertussis poliomyelitis tetanus Haemophilusinfluenzae type b hepatitis B vaccine/ or exp diphtheria pertussis poliomyelitis tetanus vaccine/ or exp Bordetella pertussis/ or exp diphtheria pertussis tetanus Haemophilusinfluenzae type b hepatitis B vaccine/

35. exp herpes simplex/

36. exp Enterovirus 71/ or exp Enterovirus infection/ or exp Enterovirus/

37. expParechovirus/ or exp virus infection/

38. exp Respiratory syncytial pneumovirus/ or exp respiratory tract infection/ or exp virus infection/ or exp viral respiratory tract infection/

39. exp candidiasis/ or exp central nervous system infection/

40. exp urinary tract infection/

41. exp Human immunodeficiency virus/

42. exp acquired immune deficiency syndrome/

43. 1 or 2 or 3 or 4 or 5 or 6 or 7 or 8 or 9 or 10 or 11 or 12 or 13 or 14 or 15 or 16 or 17 or 18 or 19 or 20 or 21 or 22 or 23 or 24 or 25 or 26 or 27 or 28 or 29 or 30 or 31 or 32 or 33 or 34 or 35 or 36 or 37 or 38 or 39 or 40 or 41 or 42

44. exp neonatology/

45. newborn/

46. exp prematurity/ or exp gestational age/

47. 44 or 45 or 46

48. 43 and 47

49. limit 48 to (human and yr="2011" and infant )

GAIA MEDLINE run 9/7/2015

1. *infection/ or exp hospital infection/ or exp bloodstream infection/ or exp bacteremia/ or exp catheter infection/

2. exp virus infection/ or *infection/

3. exp septic shock/

4. exp staphylococcal bacteremia/ or exp bacteremia/

5. exp septicemia/ or exp hemorrhagic septicemia/ or exp Viral hemorrhagic septicemia virus/ or exp viral hemorrhagic septicemia/

6. exp bacterial infection/

7. exp central nervous system infection/ or exp bacterial meningitis/

8. *infection/ or exp meningoencephalitis/ or expmeningococcosis/ or exp meningitis/

9. exp meningoencephalitis/

10. exp fungal meningitis/ or exp pneumococcal meningitis/ or exp group B streptococcal meningitis/ or meningitis/ or exp Candida meningitis/ or expHaemophilus meningitis/ or exp bacterial meningitis/ or exp aseptic meningitis/ or exp meningitis vaccine/ or exp virus meningitis/ or exp epidemic meningitis/

11. exp omphalitis/

12. exp brain ventriculitis/

13. exp group B streptococcal pneumonia/ or exp ventilator associated pneumonia/ or exp lobar pneumonia/ or exp Pseudomonas pneumonia/ or exp chlamydial pneumonia/ or exp health care associated pneumonia/ or exp infectious pneumonia/ or exp virus pneumonia/ or exp hospital acquired pneumonia/ or exp bacterial pneumonia/ or exp Streptococcus pneumonia/ or exp pneumonia/ or exp staphylococcal pneumonia/ or exp Escherichia coli pneumonia/

14. exp chest infection/

15. exp bronchiolitis/ or exp viral bronchiolitis/

16. exp osteomyelitis/

17. exp osteomyelitis/ or exp infectious arthritis/ or exp bone infection/ or exp soft tissue infection/

18. exp bacterial arthritis/

19. exp hospital infection/

20. exp urinary tract infection/ or exp Staphylococcus infection/

21. exp vertical transmission/ or exp newborn infection/ or exp Streptococcus infection/ or exp Streptococcus agalactiae/

22. exp Escherichia coli/

23. exp methicillin resistant Staphylococcus aureus/

24. exp Staphylococcus aureus/

25. exp Enterobacter aerogenes/ or exp Enterobacter/ or exp "Enterobacter cloacae subsp. dissolvens"/ or exp Enterobacter cloacae/

26. exp Acinetobacter junii/ or exp Acinetobacter baumannii/ or exp Acinetobacter infection/ or exp Acinetobacter/ or exp Acinetobacter calcoaceticus/ or exp Acinetobacter lwoffii/ or exp Acinetobacter baylyi/ or exp Acinetobacter johnsonii/

27. expKlebsiella pneumoniae infection/ or expKlebsiella vaccine/ or expKlebsiella/ or expKlebsiellaoxytoca/ or expKlebsiella infection/ or expKlebsiella pneumoniae/

28. exp Pseudomonas infection/ or exp Pseudomonas aeruginosa/ or exp Pseudomonas vaccine/ or exp Pseudomonas/ or exp Pseudomonas stutzeri/

29. expSerratia infection/ or expSerratia/ or expSerratiamarcescens/ or expSerratialiquefaciens/

30. exp Listeria monocytogenes/ or exp Listeria/

31. exp Staphylococcus aureus/ or exp bacteremia/ or exp bacterial infection/ or exp coagulase negative Staphylococcus/ or exp hospital infection/ or exp surgical infection/ or exp catheter infection/ or exp Staphylococcus infection/

32. exp bronchiolitis/ or exp respiratory syncytial virus infection/ or exp Respiratory syncytial pneumovirus/ or *infection/

33. exp Human cytomegalovirus/ or exp Cytomegalovirus/ or exp cytomegalovirus infection/ or exp Cytomegalovirus vaccine/

34. exp diphtheria pertussis tetanus Haemophilusinfluenzae type b vaccine/ or exp pertussis vaccine/ or exp diphtheria pertussis poliomyelitis tetanus hepatitis B vaccine/ or exp diphtheria pertussis tetanus hepatitis B vaccine/ or exp pertussis/ or exp diphtheria pertussis poliomyelitis tetanus Haemophilusinfluenzae type b vaccine/ or exp diphtheria pertussis tetanus vaccine/ or exp diphtheria pertussis poliomyelitis tetanus Haemophilusinfluenzae type b hepatitis B vaccine/ or exp diphtheria pertussis poliomyelitis tetanus vaccine/ or exp Bordetella pertussis/ or exp diphtheria pertussis tetanus Haemophilusinfluenzae type b hepatitis B vaccine/

35. exp herpes simplex/

36. exp Enterovirus 71/ or exp Enterovirus infection/ or exp Enterovirus/

37. expParechovirus/ or exp virus infection/

38. exp Respiratory syncytial pneumovirus/ or exp respiratory tract infection/ or exp virus infection/ or exp viral respiratory tract infection/

39. exp candidiasis/ or exp central nervous system infection/

40. exp urinary tract infection/

41. exp Human immunodeficiency virus/

42. exp acquired immune deficiency syndrome/

43. 1 or 2 or 3 or 4 or 5 or 6 or 7 or 8 or 9 or 10 or 11 or 12 or 13 or 14 or 15 or 16 or 17 or 18 or 19 or 20 or 21 or 22 or 23 or 24 or 25 or 26 or 27 or 28 or 29 or 30 or 31 or 32 or 33 or 34 or 35 or 36 or 37 or 38 or 39 or 40 or 41 or 42

44. exp neonatology/

45. newborn/

46. exp prematurity/ or exp gestational age/

47. 44 or 45 or 46

48. 43 and 47

49. limit 48 to (english language and humans and yr="2011 -Current" and "all infant (birth to 23 months)" and english and humans and medline and last 5 years)

GAIA Neonatal Infections COCHRANE, RUN 10/09/2015

#1 MeSH descriptor: [Infant] explode all trees

#2 MeSH descriptor: [Infant, Low Birth Weight] explode all trees

#3 MeSH descriptor: [Infant, Premature] explode all trees

#4 MeSH descriptor: [Bacteremia] explode all trees

#5 MeSH descriptor: [Sepsis] explode all trees

#6 MeSH descriptor: [Systemic Inflammatory Response Syndrome] explode all trees

#7 MeSH descriptor: [Sepsis] explode all trees

#8 MeSH descriptor: [Bacterial Infections] explode all trees

#9 MeSH descriptor: [Central Nervous System Infections] explode all trees

#10 MeSH descriptor: [Meningitis] explode all trees

#11 MeSH descriptor: [Meningitis, Viral] explode all trees

#12 MeSH descriptor: [Meningitis, Aseptic] explode all trees

#13 MeSH descriptor: [Meningitis, Bacterial] explode all trees

#14 MeSH descriptor: [Meningitis, Fungal] explode all trees

#15 MeSH descriptor: [Cerebral Ventriculitis] explode all trees

#16 MeSH descriptor: [Pneumonia] explode all trees

#17 MeSH descriptor: [Bronchiolitis] explode all trees

#18 MeSH descriptor: [Osteomyelitis] explode all trees

#19 MeSH descriptor: [Arthritis, Infectious] explode all trees

#20 MeSH descriptor: [Malaria] explode all trees

#21 MeSH descriptor: [Tetanus] explode all trees

#22 MeSH descriptor: [Haemophilusinfluenzae] explode all trees

#23 MeSH descriptor: [Cross Infection] explode all trees

#24 MeSH descriptor: [Streptococcus agalactiae] explode all trees

#25 MeSH descriptor: [Streptococcus pyogenes] explode all trees

#26 MeSH descriptor: [Methicillin-Resistant Staphylococcus aureus] explode all trees

#27 MeSH descriptor: [Staphylococcus aureus] explode all trees

#28 MeSH descriptor: [Escherichia coli] explode all trees

#29 MeSH descriptor: [Enterobacteriaceae] explode all trees

#30 MeSH descriptor: [Acinetobacter] explode all trees

#31 MeSH descriptor: [Enterobacter] explode all trees

#32 MeSH descriptor: [Klebsiella] explode all trees

#33 MeSH descriptor: [Klebsiella Infections] explode all trees

#34 MeSH descriptor: [Pseudomonas] explode all trees

#35 MeSH descriptor: [Serratia] explode all trees

#36 MeSH descriptor: [Listeria] explode all trees

#37 MeSH descriptor: [Respiratory Syncytial Virus, Human] explode all trees

#38 MeSH descriptor: [Cytomegalovirus] explode all trees

#39 MeSH descriptor: [Bordetella pertussis] explode all trees

#40 MeSH descriptor: [Herpes Simplex] explode all trees

#41 MeSH descriptor: [Enterovirus] explode all trees

#42 MeSH descriptor: [Parechovirus] explode all trees

#43 MeSH descriptor: [Influenza, Human] explode all trees

#44 MeSH descriptor: [Metapneumovirus] explode all trees

#45 MeSH descriptor: [Bocavirus] explode all trees

#46 MeSH descriptor: [Viruses] explode all trees

#47 MeSH descriptor: [Urinary Tract Infections] explode all trees

#48 MeSH descriptor: [Bacteria] explode all trees

#49 MeSH descriptor: [HIV] explode all trees

#50 MeSH descriptor: [Fungi] explode all trees

#51 #1 or #2 or #3 Publication Year from 2011 to 2015

#52 #4 or #5 or #6 or #7 or #8 or #9 or #10 or #11 or #12 or #13 or #14 or #15 or #16 or #17 or #18 or #19 or #20

#53 #21 or #22 or #26 or #27 or #28 or #29 or #30 or #31 or #32 or #23 or #24 or #25 or #33 or #34 or #35

#54 #36 or #37 or #38 or #39 or #40 or #41 r or #42 or #43 or #44 or #45 or #46 or #47 or #48 or #49 or #50

#55 #52 or #53 or #54 Publication Year from 2011 to 2015

#56 #55 and #51 Publication Year from 2011 to 2015
