# Supplementary material for: Neonatal infections: Case definition and guidelines for data collection, analysis, and presentation of immunisation safety data
Source: Vaccine. 2016 Dec 1;34(49):6038–46. doi: 10.1016/j.vaccine.2016.03.046 (PMC5139809; doi:10.1016/j.vaccine.2016.03.046)
Supplement: Supplementary file 2 [file mmc2.docx]

| **Recognised pathogens** | **Non pathogens (but may be opportunistic pathogens in the neonatal period)** |
| --- | --- |
| **Bacteria** | **Bacteria** |
| ***Acinetobacter spp:***  *Acinetobacter baumannii*  *Acinetobacter lwoffii*  *Bacillus cereus*  ***Bordetella sp***  *Bordetella bronchiseptica*  *Bordetella parapertussis*  *Bordetella pertussis*  *Burkholderiacepacia*  ***Citrobacter sp****.*  *CitrobacterKoseri*  *Citrobacterdiversus*  *Citrobacterfreundii*  ***Clostridium sp.***  *Clostridium difficile*  *Clostridium perfringens*  *Eikenellacorrodens*  ***Enterococcus spp.***  *Enterococcus faecalis*  *Enterococcus faecium*  *Enterococcus gallinarum*  ***Enterobacter sp****.*  *Enterobacter aerogenes*  *Enterobacter agglomerans*  *Enterobacter cloacae*  *Escherichia coli*  ***Haemophilus sp.***  *Haemophilusinfluenzae*  *Haemophilusparainfluenzae*  ***Klebsiella sp****.*  *Klebsiellaaerogenes*  *Klebsiellaoxytoca*  *Klebsiellapneumonia*  *Lactobacillus sp.*  *Listeria monocytogenes*  ***Mycobacterium sp****.*  *Mycobacterium tuberculosis*  *Morganellamorganii*  ***Neisseria sp****.*  *Neisseria meningitidis*  *Neisseria gonorrhoeae*  ***Nocardiasp***  *Nocardiaasteroides*  ***Pantoeasp***  *Plesiomonasshigelloides*  ***Proteus sp.***  *Proteus mirabilis*  *Proteus vulgaris*  ***Providencia sp***  *Providencia rettgeri*  *Providencia stuartii*  ***Pseudomonas sp***  *Pseudomonas aeruginosa*  *Pseudomonas cepacia*  *Pseudomonas stutzeri*  *Salmonella sp.*  ***Shigella sp****.*  *Shigelladysenteriae*  *Shigellaflexneri*  *Shigellasonnei*  ***Serratia sp.***  *Serratialiquefaciens*  *Serratiamarcescens*  *Staphylococcus aureus (Methicillin-sensitive and -resistant MSSA or MRSA)*  *Streptococcus agalactiae or group B streptococcus*  *Streptococcus pneumoniae*  *Streptococcus pyogenes*  *Stenotrophomonasmaltophilia*  *Ureaplasma sp.* | *Bacillus sp. (other than B. cereus)*  ***Bacteroides sp****.*  *Bacteroidesfragilis*  Coagulase-negative staphylococci   - Coagulase-negative staphylococci (mixed)   *Corynebacterium sp.*  Diphtheroids  *Micrococcus sp.*  ***Propionibacterium sp.***  *Propionibacterium acnae*  ***Peptococcus sp.***  ***Peptostreptococcus sp****.*  *Peptostreptococcusmagnus*  *Peptostreptococcus micros*  ***Streptococcus sp****.*  *Streptococcus acidominimus*  *Streptococcus anginosus*  *Streptococcus bovis*  *Staphylococcus capitis*  *Streptococcus constellatus*  *Staphylococcus epidermidis*  *Streptococcus equinus*  *Staphylococcus haemolyticus*  *Staphylococcus hominis*  *Streptococcus mitis*  *Streptococcus mutans*  *Streptococcus oralis*  *Streptococcus salivarius*  *Streptococcus sanguis*  *Staphylococcus saprophyticus*  *Streptococcus suis*  *Streptococcus viridans*  *Staphylococcus warneri*  *Stomatococcusmucilaginosus* |
| - ***Fungi*** |  |
| ***Aspergillus spp***   - 1. *Aspergillus flavus*   2. *Aspergillus fumigatus*   3. *Aspergillus glaucus*   4. *Aspergillus niger*   5. *Aspergillus terreus*   *Aspergillus versicolor*  ***Candida sp.***   - 1. *Candida albicans*   2. *Candida dubliniensis*   3. *Candida glabrata*   4. *Candida guilliermondii*   5. *Candida kefyr*   6. *Candida krusei*   7. *Candida parapsilosis*   *Candida tropicalis* |  |
| ***Cryptococcus sp.***  *Cryptococcus neoformans* |  |
| - **Protozoa** |  |
| *Plasmodium falciparum*  *Plasmodium knowlesi*  *Plasmodium malariae*  *Plasmodium ovale*  *Plasmodium vivax*  *Toxoplasma gondii*  *Trypanosoma cruzii* |  |
| - **Viruses** |  |
| Adenovirus  Bocavirus  Coronavirus  Enteroviruses  Herpes simplex viruses  Human metapneumovirus  Influenza  Parainfluenza  Parechoviruses  Parvovirus  Respiratory Syncytial Virus  Rhinovirus  Varicella zoster virus |  |
