# Supplementary material for: Neonatal infections: Case definition and guidelines for data collection, analysis, and presentation of immunisation safety data
Source: Vaccine. 2016 Dec 1;34(49):6038–46. doi: 10.1016/j.vaccine.2016.03.046 (PMC5139809; doi:10.1016/j.vaccine.2016.03.046)
Supplement: Supplementary file 3 [file mmc3.docx]

Rebuttal - Manuscript title:Neonatal Infections: Case Definition & Guidelines for Data Collection, Analysis, and Presentation of Immunisation Safety Data

Date: 9 December 2015

|  | **Review comments** | | **Author reply** |
| --- | --- | --- | --- |
| **1.a. Do you agree with the rationale of the case definition?** | Yes | 17 |  |
|  | No | 1 |  |
|  | Do not know | 0 |  |
| 1.b. If no / do not know, explain why. | Inclusion of cnogenital infections in the definition may not be papripriate since this is a source rather than a distinct syndrome. Similarly "sepsis" may lead to any of the syndromes described, pneumonia, meningitis etc. | | We agree with this and have now removed congenital infections from the definition. |
| 1.c. Comments? | other than first page being blank, the report is well written. | | Thank you. |
|  | there are some texts missing and a few minor issues. | | Noted and corrected. |
|  | several levels were necessary because of the frequent lack of microbiological confirmation | | Agreed. |
|  | Global deaths from neonatal infections and sepsis were estimated to be 346.4 thousands and 366.0 thousands in 1990 and 2013 respectively, with no statistically significant change at 0.05 alpha level. Global age-standardized death rates per 100,000 were 4.7 and 4.9 in 1990 and 2013 respectively. The most common clinical types of fatal neonatal infections (an their estimated number of deaths in 2013 in thousands) were lower respiratory infections (196.5), diarrhoeal diseases (44.8), tetanus (26.0), meningitis (2.6), and malaria (16.8). [PMID: 25530442] | | Thank you and now included. |
| **2.a. Does the preamble clearly describe the intended use of the definition?** | Yes | 15 |  |
|  | No | 1 |  |
|  | Do not know | 0 |  |
| 2.b. If no / do not know, explain why. | On page 3, it is written, "...the GAIA neonatal infections group has developed separate case definitions for neonatal blood stream infections, meningitis, and respiratory tract infections..." however, there are case definitions provided for neonatal tetanus and congenital infections in the case definition section. While there are two small paragraphs at the end of the preamble that describe the utility of providing definitions for these two topics, it would be beneficial to clearly state this in the preamble as there is intent to use these additional case definitions. | | Agreed and as above the definitions for congenital infections have now been removed. |
|  | Seems incomplete with blanks or options | | noted |
| 2.c. Comments? | Parallel structure throughout the manuscript would greatly improve readability. | | Sorry, do not understand the suggestion! |
|  | this is more comprehensive than the other two sections.  page 13 -- table is incomplete | | noted |
|  | 1.ON P.2, PREAMBLE, 1ST PARA: LINE 5, THE PATTERN OF NEONATAL INFECTIONS COULD BE PART OF BOTH VACCINATION AE'S AND PROGRAM EFFECTIVENESS (i.e. reduced incidence due to vaccine program).  p.3, 3rd para up from page bottom: what about encephalitis, renal and intestinal infections?'  2.Ibid, 2nd para from bottom, birth should NOT be "day 0". Birth is TIME ZERO; it does not cover a whole day.  3.p.5, para2: Indicate if search confined only to articles in English.  4.p.6, top line. Define "SV".  5.P.6, bottom, pathophysiology sections: are these going to be filled in? Why, exactily? | | Agreed and expanded.  Agreed and noted.  The conventional definition of day 0, familiar to demographers, is the time from birth to 24 hours of age. This definition is widely used in the neonatal literature e.g. *Lancet Glob Health* 2014; 2: e635–44  Yes it does. Noted.  Replaced with „“by the coordinator (SV)“. SV is Stefi Vergnano.  No. now removed. |
| **2(2).a. Do you agree with the definition?** | Yes | 0 |  |
|  | No | 1 |  |
|  | Do not know | 0 |  |
| 2(2).b. If no / do not know, explain why. | In practice I foresee two areas where this will run into practical problems potentially: - coagneg staphylococci are obviously the commonest cause of late-onset sepsis in preterm infants. Most centres (at least outside the US) do not take two blood cultures and will diagnose and treat CoNS sepsis on the basis of one BC, a CRP rise and one or more clinical features.  Secondly the definition of meningitis is pretty vague (presumably deliberately given this is a bit of a minefield). However there are some data to help guide a definition of CSF pleocytosis in the absence of a positive CSF culture, as this is the most frequent clinical scenario; most infants receive antibiotics prior to CSF being obtained. There is the secondary issue of how NICUs and paediatricians differ in interpreting CSF cell counts in the presence of a blood-stained CSF. | | This point is well made. The definition for level 1 infection for what are often skin commensals is most difficult. The current definition is: „When bacteria that are considered to be skin commensals are isolated, level 1 of evidence requires at least 2 blood cultures taken from two different sites or at 2 different times plus 1 of the criteria as per level 2 of evidence“. We believe that in the context of a clinical trial the need for 2 blood cultures will be enforced by the protocol.  Agreed. However, we have subsequently modified the definition to account for this. We have also included a comment regarding interpretation of blood-stained CSF. |
| 2(2).c. Comments? | As above | |  |
| **3.a. Do you agree with Level 1 of diagnostic certainty?** | Yes | 12 |  |
|  | No | 1 |  |
|  | Do not know | 3 |  |
| 3.b. If no / do not know, explain why. | not enough details on evidence behind it | | Now further justified in the text. |
|  | It does not appear that these have been completely written in the current draft.   - On page 20 for Level 1 diagnostic certainty for RTI, it is possible that a viral pneumonia may also show an infiltrate on CXR. I would modify the case definition to account for this. | | Agree. Now completed.  Agreed, however an infiltrate on CXR is not required to allow a level 1 case definition for viral LRTI. It is however necessary to capture a level 1 case definition for a presumed bacterial LRTI. |
|  | 1.This section is muddy. Where is level 2??  2.What is meant by "microbiological"? Does this mean culture isolation? What about antibody levels and changes in those levels? PCR?  3.P.8, para 3: what is supposed to be after the end ("differentiation based on....."??? | | This has been updated subsequently.  Yes, refers to isolate or identification via culture or molecular methods. Antibody levels have not been included in the definitions.  This has been completed now. |
|  | Seems incomplete with blanks or options | | corrected |
|  | - Consider mentioning what is exactly meant by “a validated method” and “other validated inflammatory markers”, and which methods are not valid.   - Consider proving quantitative cutoff values for fever, hypothermia, metabolic acidosis, tachypnea, increased CRP or other validated inflammatory markers, thrombocytopenia, positive Dye test, positive blood VDRL, positive specific anti-treponemal serology (EIA or TTPA), RPR, elevated and white cell count in CSF  - Is breastfeeding exclusive breastfeeding or not. | | This is now discussed further.  Values now provided where appropriate.  HIV definition now removed. |
| 3.c. Comments? | It is unclear why there are two areas in the draft that go over the case definitions. The first, starting on page 7, which appears to be section 2 of the manuscript and is titled, "CASE DEFINITION OF NEONATAL INFECTIONS" should be where case definitions are listed. The second area focusing on case definitions, starts on page 16 and is section 3.5, entitled, "Case Definitions" then goes into further detail. This is confusing. If possible these sections should be combined. I think it would make more sense to have the case definitions listed under section 2 as some of the information discussed subsequently would be make more sense if the real case definition were presented before data analysis and presentation. | | The sections have now been combined as suggested. |
|  | for tetanus, I think the progressive nature of the disease should be part of the definition. Right now it is described as if it was static. | | Tetanus definition now removed. |
|  | May be the prsence of germs in the gastric fluid (for newborns who have not be already fed) associate to clinical signs can also be considered as level 1 | | We considered this but felt that a level 1 definition should include sterile site samples only. |
|  | The pepaer is a bit immature e.g. meninigitis definition: levels are missing; syphilis clinical signs are missing; hepatitsi, chagas disease, malaria, HIV only one level.. | | Agreed and now updated where appropriate. |
| **4.a. Do you agree with Level 2 of diagnostic certainty?** | Yes | 12 |  |
|  | No | 2 |  |
|  | Do not know | 2 |  |
| 4.b. If no / do not know, explain why. | see above | |  |
|  | Not all case definitions have level 2 of diagnostic certainty listed. On page 21 for the level 2 diagnostic certainty for CMV infections, requiring only two clinical criteria for a CMV infection seems too sensitive and therefore lacks almost any specificity. For instance, a neonate with transaminitis and HSM would be labeled as having a CMV infection. This constellation of symptoms could also represent HSV, inborn errors of metabolism, viral hepatitis, and a number of other infections. I would strongly recommend the work group go through each of the definitions to ensure each level of diagnostic certainty affords the appropriate balance of sensitivity and specificity. | | The congenital infection definitions have now been removed. |
|  | Couldn't find a level 2. | | ? |
|  | Seems incomplete with blanks or options | | corrected |
| 4.c. Comments? | jaundice could be add to the clinical criteria as it can be a sign of hemolysis even if it is not so specific. | | We did not find sufficient evidence to include jaundice as a criterion. |
| **5.a. Do you agree with Level 3 of diagnostic certainty?** | Yes | 10 |  |
|  | No | 2 |  |
|  | Do not know | 3 |  |
| 5.b. If no / do not know, explain why. | see above | |  |
|  | These were not provided for any of the case definitions as far as I can tell, but maybe they are not showing up on my computer while viewing the PDF? | | Now updated |
|  | not applicable | | As above |
|  | No clear level 3 given. | | As above |
|  | Seems incomplete with blanks or options | | Corrected |
| 5.c. Comments? |  | |  |
| **6.a. Do you agree with the footnotes of the case definition?** | Yes | 14 |  |
|  | No | 1 |  |
|  | Do not know | 1 |  |
| 6.b. If no / do not know, explain why. | There are some footnotes that are marked but then no footnote is provided (see pg. 19, Bacterial / fungal / viral meningitis). Some of the info in the footnotes should be included in the definitions themselves (pg. 18, explanation of when a skin contaminant should be considered as a level 1 diagnostic certainty for a systemic infection should be more clear and not within a footnote). Please use the same terminology within footnotes and appendices - (e.g., skin commensals vs skin contaminants). | | This has been corrected.  Agree. This has now been included in the main table.  Corrected. |
|  | Seems incomplete with blanks or options - and maybe not applicable in some cases | | Corrected. |
| 6.c. Comments? |  | |  |
| **7.a. Is the definition applicable in your setting?** | Yes | 13 |  |
|  | No | 0 |  |
|  | Do not know | 3 |  |
| 7.b. If no / do not know, explain why. | need more information on background to definition (ie level of evidence) | | More information now included |
|  | The definition needs to be fully expanded in order to make this determination. | | Now expanded |
|  | No clear level definitions in text. Appear too late on pp.16 et seq. Level 1 should include SOME clinical signs | | The WG discussed this but felt that if a significant pathogen (e.g. Group B streptococcus) was isolated from a normally sterile site (e.g. the blood), then this should be sufficient to meet a level 1 definition, even in the absence of clinical signs. |
| 7.c. Comments? | In the US, we would probably do more genetic and DNA testing for infectious agent typing the strain or variant, but this is not applicable to LMIC | | Agreed but we hope that we have made the definitions suitable for all settings. |
|  | even if we rarely obtain the level 1 of certainly because of the technical insufficiency | |  |
|  | It is partially applicable. I am unsure if these guidelines will be applicable to community of facility setting or both. It will be challenging to implement in the community. That's my initial take. | | Changed: The Levels 2 and particularly 3 definitions have been discussed with the idea that community studies in LMIC could also be able to use them |
| **8.a. Do you agree with the guidelines?** | Yes | 12 |  |
|  | No | 2 |  |
|  | Do not know | 3 |  |
| 8.b. If no / do not know, explain why. | my impression was that this was a work in progress rather than a finished product | | Agreed! |
|  | Incomplete guideline which needs further revisions. | | Agreed! |
|  | 1.Very overelaborate and mindbogglingly confusing.  Best deleted and replaced with a clear table.  2.P. 13 can't review with no times filled in. | | Now updated. |
|  | Seems incomplete with blanks or options | | Agreed and now complete. |
|  | largely, but reservations 9which may be partly intractable) as above | | Hopefully addressed above |
| 8.c. Comments? | pp.21 et seq. : Could not find any logic to presentation, which bounces from virus to parasite to bacteria to virus etc etc.  On p.23, bottom: should be: Definitions still TO BE added. Paper is too rough to justify review at this stage.PP. 24 et seq: Heading for "contaminants" should always say "most probably contaminants", as sometimes some of these organisms are pathogens. | | Now removed.  Done now.  Noted but to be considered so they need to have additional evidence, as detailed. |
|  | as above | |  |
| **9. Any general comments?** | Clear and concise | | thanks |
|  | should aim at minimal information needed rather than ideal. what is necessary to make a decision about the vaccine safety. information on vaccination (ie how give and into what area) question relevance as it stands. what evidence is that all the details are needed.  My concern is when asking to much from investigators or persons reporting adverse reactions you will end up with a lot of missing information or information leading to misclassification | | Agreed. The information required on vaccination and on adverse events is covered in the guidelines document. |
|  | - Should references be listed within or outside of punctuation? - I disagree with the decision to exclude case reports. It would be beneficial for those who write case reports to use the definitions. - There could be more discussion about the use of the case definitions in different contexts - LMIC vs high income countries. - On pg. 11, "25) Objective clinical evidence supporting classification of the event as 'serious'" - a definition of what is acceptable as evidence for classifying an event as serious should be provided. Additionally, on page 15, it is written, "Terms to describe neonatal infection such as “low‐grade”, “mild”, “moderate”, “severe” or “significant” are highly subjective, prone to wide interpretation, and should be avoided, unless clearly defined." How do you reconcile these two statements?  - Page 18 has "metabolic acidosis" listed as part of the level 2 diagnostic certainty for systemic infection. There should be a footnote defining how a metabolic acidosis is defined - need for ABG? VBG or ABG? BMP only? | | Updated.  Comment noted but this was decided early in the process.  Further discussion now included.  Serious is defined in a footnote (7).  These terms have different significance to the term „serious“ which has an agreed set of criteria as indicated in the footnote.  Now defined. |
|  | This case definition will be very useful in my seeting were newborns are often considered as infected without any pertinent criteria | |  |
|  | The tables of pathogens have no logic and are in no apparent order, alphabetical or otherwise. A mishmash.  Some important congenital infections are absent, such as rubella.  On p.31, bottom, country names should be capitalized.  The references have variable numbers of authors listed. Should confirm to journal requirements.  Appendix A missing. | | The list of commensals is now alphabetical.  The congenital infection definitions have now been removed.  ?  Done  Now included |
|  | This Guidelines are very useful so a wide spread for health staff will be necessary. | | Thank you |
|  | The various case definitions could be tabulated and compared The other minor issue is that the manuscript is getting little longer and has potential to edit and reduce the text. | | Agreed and done. |
|  | the guideline has taken into consideration a definition applicable to the LMIC situation | | We hope this is now applicable |
|  | Being a Neonatologist I am involved with diagnosis of neonatal infections routinely and utilize all of the definitions outlined in this manuscript. Very detailed manuscript noting the importance of both clinical and diagnostic possibilities. | | Thank you. |
| **7. Any other comments you would like to share?** |  | |  |
| **3.a. Based on your study setting and the procedures / diagnostic requirements for the different levels of certainty in the case definition, would you be able to obtain all levels of certainty?** | Yes | 0 |  |
|  | No | 0 |  |
|  | Do not know | 1 |  |
| 3.b. If no / do not know, explain which level(s) would not be applicable and why (e.g. not having all procedures available in countries where study is done / retrospective assessment of charts would not allow information / exclusion / negative criteria to be found consistently) | as above | |  |
| 3.c. Comments? | as above | |  |
